# Circadian Rhythm Alteration of the Core Clock Genes and the Lipid Metabolism Genes Induced by High-Fat Diet (HFD) in the Liver Tissue of the Chinese Soft-Shelled Turtle (*Trionyx sinensis*)

**DOI:** 10.3390/genes15020157

**Published:** 2024-01-25

**Authors:** Li Liu, Lingli Liu, Shiming Deng, Li Zou, Yong He, Xin Zhu, Honghui Li, Yazhou Hu, Wuying Chu, Xiaoqing Wang

**Affiliations:** 1 School of Medical Technology, Shaoyang University, Shaoyang 422000, China; hnhhliliu@163.com; 2 Fisheries Research Institute of Hunan Province, Changsha 410153, China; 13507477871@163.com (L.L.); dsmjgm@126.com (S.D.);; 3 College of Biological and Chemical Engineering, Changsha University, Changsha 410003, Chinalee19890925@163.com (H.L.); 4 Fisheries College, Hunan Agriculture University, Changsha 410128, China; huyazhou@hunau.edu.cn

**Keywords:** *Trionyx sinensis*, high-fat diet, core clock genes, lipid metabolism genes, circadian rhythm, correlation

## Abstract

Physiology disorders of the liver, as it is an important tissue in lipid metabolism, can cause fatty liver disease. The mechanism might be regulated by 17 circadian clock genes and 18 fat metabolism genes, together with a high-fat diet (HFD). Due to their rich nutritional and medicinal value, Chinese soft-shelled turtles (*Trionyx sinensis*) are very popular among the Chinese people. In the study, we aimed to investigate the influence of an HFD on the daily expression of both the core clock genes and the lipid metabolism genes in the liver tissue of the turtles. The two diets were formulated with 7.98% lipid (the CON group) and 13.86% lipid (the HFD group) to feed 180 juvenile turtles, which were randomly divided into two groups with three replicates per group and 30 turtles in each replicate for six weeks, and the diet experiment was administrated with a photophase regimen of a 24 h light/dark (12L:12D) cycle. At the end of the experiment, the liver tissue samples were collected from nine turtles per group every 3 h (zeitgeber time: ZT 0, 3, 6, 9, 12, 15, 18, 21 and 24) for 24 h to investigate the daily expression and correlation analysis of these genes. The results showed that 11 core clock genes [i.e., circadian locomotor output cycles kaput (*Clock*), brain and muscle arnt-like protein 1 and 2 (*Bmal*1/2), timeless (*Tim)*, cryptochrome 1 (*Cry*2), period2 (*Per*2), nuclear factor IL-3 gene (*Nfil*3), nuclear receptor subfamily 1, treatment D, member 1 and 2 (*Nr1d*1/2) and retinoic acid related orphan receptor α/β/γ *β and γ* (*Rorβ*/*γ*)] exhibited circadian oscillation, but 6 genes did not, including neuronal PAS domain protein 2 (*Npas*2), *Per*1, *Cry*1, basic helix-loop-helix family, member E40 (*Bhlhe*40), *Rorα* and D-binding protein (*Dbp*), and 16 lipid metabolism genes including fatty acid synthase (*Fas*), diacylglycerol acyltransferase 1 (*Dgat*1), 3-hydroxy-3-methylglutaryl-CoA reductase (*Hmgcr*), Low-density lipoprotein receptor-related protein 1-like (*Ldlr*1), Lipin 1 (*Lipin*1), Carnitine palmitoyltransferase 1A (*Cpt*1a), Peroxisome proliferator activation receptor α, β and γ (*Pparα*/*β*/*γ*), Sirtuin 1 (*Sirt*1), *Apoa* (*Apoa*1), Apolipoprotein B (*Apob*), Pyruvate Dehydrogenase kinase 4 (*Pdk*4), Acyl-CoA synthase long-chain1 (*Acsl*1), Liver X receptors α (*Lxrα*) and Retinoid X receptor, α (*Rxra*) also demonstrated circadian oscillations, but 2 genes did not, *Scd* and *Acaca,* in the liver tissues of the CON group. However, in the HFD group, the circadian rhythms’ expressional patterns were disrupted for the eight core clock genes, *Clock*, *Cry*2, *Per*2, *Nfil*3, *Nr*1*d*1/2 and *Rorβ*/*γ*, and the peak expression of *Bmal*1/2 and *Tim* showed delayed or advanced phases. Furthermore, four genes (*Cry*1, *Per*1, *Dbp* and *Rorα*) displayed no diurnal rhythm in the CON group; instead, significant circadian rhythms appeared in the HFD group. Meanwhile, the HFD disrupted the circadian rhythm expressions of seven fat metabolism genes (*Fas*, *Cpt*1a, *Sirt*1, *Apoa*1, *Apob*, *Pdk*4 and *Acsl*1). Meanwhile, the other nine genes in the HFD group also showed advanced or delayed expression peaks compared to the CON group. Most importantly of all, there were remarkably positive or negative correlations between the core clock genes and the lipid metabolism genes, and their correlation relationships were altered by the HFD. To sum up, circadian rhythm alterations of the core clock genes and the lipid metabolism genes were induced by the high-fat diet (HFD) in the liver tissues of *T. sinensis*. This result provides experimental and theoretical data for the mass breeding and production of *T. sinensis* in our country.

## 1. Introduction

Most organisms display a 24 h circadian rhythm, which is closely bound up with the periodic changes of the earth and the sun. The physiological and behavioral rhythms are controlled and regulated by an endogenous timekeeping system (named the circadian clock) to make it suitable for the environment [1,2]. However, the circadian clock is composed of two parts, including both the master clock and the peripheral clock, and the former is located in the central region (the suprachiasmatic nucleus of the hypothalamus, SCN), and the latter is scattered among the peripheral tissues such as kidney, heart, liver, skeletal muscle, etc. [3]. Recent studies have revealed that circadian rhythms are caused by a biological oscillation, which is performed by a transcriptional translational feedback loop. The loop contains both the positive arm and the negative arm, and the positive arm is involved with a set of the core clock genes including encoding transcription-activated protein factors such as circadian locomotor output cycles kaput (CLOCK), brain and muscle Arnt-like proteins 1 and 2 (BMAL1/2) and neuronal PAS domain protein 2 (NPAS2), and the negative arm is composed of the encoding translation-suppressive proteins including periods 1, 2 and 3 (PER1/2/3) and cryptochromes 1 and 2 (CRY1/2) [4,5]. Furthermore, it is also activated to express these genes, which are named the transcription factors and the nuclear receptor factors, including D-binding protein (*Dbp*), basic helix–loop–helix family, member E40 (*Bhlhe*40), nuclear factor IL-3 gene (*Nfil*3), retinoic acid-related orphan receptors α, β and γ (*Rorα*/*β*/*γ*) and nuclear receptor subfamily 1, treatment D, members 1 and 2 (*Nr*1*d*1/2, also called Rev-erbα/β), by the two genes, *Clock* and *Bmal*1 [6,7,8,9]. It has also been indicated that the diseases of sleep disorders, metabolic chaos and other symptoms can occur via the alterations of both energy metabolism and circadian rhythms [10].

It is well known that there is an internal timing system (i.e., the circadian clock) in the rodent liver, with major metabolic hub–hepatic functions such as nutrient metabolism, detoxification and synthesis that is controlled by 8–15% of rhythmically expressed genes [11,12]. The physiological activities of the liver are accomplished through these factors, including metabolic nuclear receptors, and the metabolic enzymes, which are regulated by the core circadian clock, and these factors in return control the core circadian clock to formulate the cyclic expression [10]. For example, 3-hydroxy-3-methylglutaryl-CoA reductase (*Hmgcr*), as a rate-limiting enzyme in cholesterol biosynthesis, had the highest activity at night via the regulation of the core circadian clock [13]. Studies reported that there was a positive correlation relationship between the expressions of two genes, the *Clock* gene and the lipoprotein lipase (LPL) gene, which were the target of peroxisome proliferator-activated receptor α (*Pparα*), and could promote the decomposition of TGs (triglycerides) [14]. sirtuin 1 (*Sirt*1), as both an acetylation enzyme that is NAD+-dependent and a key factor to regulate liver circadian rhythms, showed a circadian oscillation by entering the *Clock* gene promoter with interacting CLOCK/BMAL1 protein dimers. It was also suggested that a gene expression rheostat of the core clock in the *Sirt*1 mutant mice caused corresponding changes in both *Bmal*1 acetylation and *Per*2 deacetylation [15,16,17]. Moreover, CLOCK/BMAL1 protein dimer could be activated to express through the E-box element on the peroxisome proliferator-activated receptors α, β and γ (*Pparα*/*β*/*γ*) promoter. On the contrary, the *Pparα* transcription level was also regulated by the cis-acting element on the *Bmal*1 promoter [18]. To sum up, evidence was provided for the close relationship between the core clock genes and the lipid metabolism genes.

It was found that the core circadian clock genes played a significant role in regulating nutrition and metabolism levels via the related metabolism signals. And these genes’ expressions were also in turn affected by the signal factors from nutrient levels and metabolism [10]. A dramatic effect was observed when considering the circadian rhythm and the mRNA levels of the core circadian clock genes, the nuclear receptors and the clock-controlled genes in the hypothalamus, liver and adipose tissues of mice being fed a high-fat diet (HFD) [19]. In the process of lipid metabolism, some hormones or lipids could be recognized by the nuclear receptors, which caused the expression levels of lipid-related genes to change and ultimately adapt to the demands of the body’s energy metabolism [20]. Some research had indicated that the consequences of interaction caused by *Per*2 combined with *Pparα* and *Nr*1*d*1 further regulated *Bmal*1 gene expression, while *Pparγ* with high expression levels in fat cells played an important role in both the process of fatty acid oxidation and mediating lipid metabolism [21]. It was shown that the circadian oscillations of adiponectin signaling pathway components were severely shifted or delayed in the brain and peripheral tissues of the HFD mice for 7 weeks [22,23,24].

The Chinese soft-shelled turtle (*Trionyx sinensis*) is very popular among the Chinese people due to its being regarded as one of the water treasures and its unique qualities, with rich nutritional and medicinal value [4,25,26]. By 2022, the farming yields of *T. sinensis* in China ranked No. 1 in the world, with an annual output value in excess of CNY 50 billion [27]. However, the farmers would like to reduce feed costs and increase economic interest by usually using a high-fat diet instead of fish meal in response to the high protein demanded (more than 40%) to feed the turtles. This change could result in fatty liver disease and, further, disorders of lipid metabolism and circadian rhythm. In this study, we aimed to research the influence on the circadian rhythm mechanisms of the core clock genes and the lipid metabolism genes of an HFD in liver tissues to provide strong support for the healthy and ecological culture of *T. sinensis*.

## 2. Materials and Methods

### 2.1. Experimental Diets

The two diets were formulated according to the compositions reported by previous research from our laboratory [4]. The feed materials and proportions included white fish meal (43.0%), liver meal (5.5%), α-starch (18.0%), brewer’s yeast (10.0%), expanded soybean meal (13.0%), commercial mineral premix (2.0%) and commercial vitamin premix (2.0%) in both diets. There were different materials and proportions of wheat meal (6.5% and 0.5%) and fish oil (0 and 6.0%) in the control diet (CON) group and the high-fat diet (HFD) group, respectively. Ultimately, there were significant differences in the contents of crude fat, 7.98% and 13.86%, and non-significant differences in the contents of crude protein, 43.28% and 42.93%, crude ash (12.12% and 11.43%) and energy levels (18.35 and 20.17 KJ g^−1^) in the two diets. We used % dry matter to refer to the compositions of the feed materials and formulation products. The powdered feed products were mixed with water in a ratio of 1:1 to feed the turtles or stored in a −20 °C freezer before the feed time. 

### 2.2. Animals and Experimental Design

**Figure 1:**
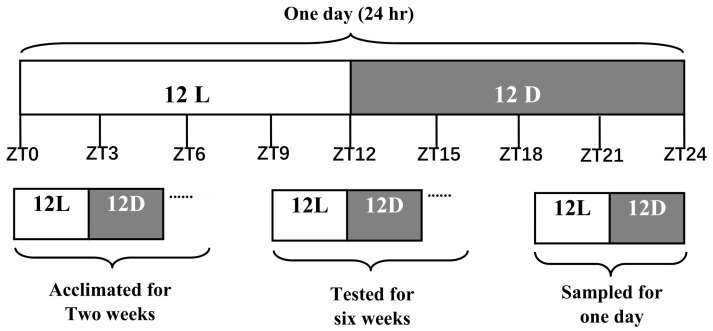
Experimental design. Note: *T. sinensis* (*n* = 180, weight = 60.0 ± 1.0 g) were acclimated for two weeks and kept at a 30 ± 1 °C range in a photoperiod of 12L:12D, and then were divided into 6 concrete tanks (30 turtles per tank) to complete the test experiment for six weeks with the same environmental conditions. After the culture experiment, the liver tissues of nine turtles per group at each zeitgeber time of ZT 0, 3, 6, 9, 12, 15, 18, 21 and 24 were collected for the detection of gene expression and circadian rhythm alteration by qRT-PCR.

The experimental animals and design were the same as described in the previous report from our laboratory [4]. That is to say, 180 juvenile turtles were obtained from the Fisheries Research Institute of Hunan Province, Changsha, China and were distributed into two diet formula groups, with fat contents of 7.98% (the CON group) and 13.86% (the HFD group) using a completely randomized design. The turtles were fed for 6 weeks under a daily photoperiod regime of 12 h light:12 h dark (12L:12D), which contained a photoperiod cycle for 24 h starting from 8 a.m. (Figure 1). 

### 2.3. Sample Collection

The method of sample collection was also as previously described [4]. Before being sacrificed by cervical dislocation, the turtles were anesthetized with anesthetics of MS-222, and then the liver tissues of 9 turtles of each group were collected at 3 h intervals (zeitgeber time, i.e., ZT 0, 3, 6, 9, 12, 15, 18, 21 and 24) starting from 8 a.m., and then rapidly immersed at −80 °C until RNA extraction. 

### 2.4. RNA Extraction and Expression by Quantitative Real-Time PCR (RT-qPCR) Analysis

The method of RNA extraction and expression analysis by quantitative real-time PCR (RT-qPCR) was reported in the previous report from our laboratory [4]. The total RNA extraction was performed by the liver tissue samples being ground in liquid nitrogen from the manufacturers of TRIzol^R^ Reagent (TaKaRa, Dalian, China), and then we used reverse transcription to obtain the cDNA products using a PrimerScript^TM^ II 1st Strand cDNA Synthesis Kit (TaKaRa, Dalian, China). The reaction system, of an amount of 10 μL, was composed of 6 μL RNase free ddH_2_O, 1 μL Oligo dT Primer, 1 μL dNTP Mixture, 2 μL total RNA, and then denatured at 65 °C for 5 min, and annealed on ice for 2 min. Then we added the reagent, including 4 μL 5× PrimeScript^TM^ II Buffer, 0.5 μL RNase Inhibitor (40 U/μL), 4.5 μL RNase free ddH_2_O, 1 μL PrimeScript^TM^ II RTase (200 U/μL), making up 20 μL of the total reaction system. The reaction was performed by a reverse transcriptional reaction procedure including incubation at 42 °C for 60 min, incubation at 70 °C for 15 min, termination at 4 °C. The cDNA products were used as templates to directly detect the expression levels of the core clock genes and fat metabolism genes by the PCR amplification method or to be stored in the refrigerator at −80 °C. Simultaneously, the negative control test was accomplished without either cDNA template or transcriptase.

The software Primer 5.0 (Premier, Winnipeg, MB, Canada). was used to design the RT-qPCR primers, whose CDS sequences of the genes were obtained from the GeneBank accession numbers of the NCBI website (Table 1). And the primers were synthesized by the Shanghai Yingjun Biology Company I. *Rpl*19 was used as the optimal reference gene for an inner control, to be suitable for the qRT-PCR method in the liver tissue, and was detected and chosen from six candidate reference genes including *Gapdh*, 18S rRNA, *Rpl*13, *Rpl*19, *Rps*2 and β-actin by using the Online software of geNorm [28], Norm Finder [29] and Bestkeeper software [30] in our laboratory. The fluorescent quantitative instrument was the Bio-Rad CFX96 system (Bio-Rad, Hercules, CA, USA); the operation procedure on the TaKaRa SYBR Premix Ex Taq^TM^ II RT-qPCR kit (TaKaRa, Dalian, China) was according to the previously described method [31]. The expression levels of the core clock genes and the lipid metabolism genes were quantified through an RT-qPCR quantification reaction. The reaction system of 25 µL was added in the form of 1 µL cDNA template, 0.5 µL of gene-specific F/R primer (10 mmol/L), 12.5 mL SYBR Green mix and 10.5 mL RNase-free ddH_2_O, and then the procedure was executed for 39 circulations, including the 4 steps of predenaturation at 95 °C for 1 min, denaturation at 95 °C for 5 s, annealing at 56~61 °C for 30 s, extension at 72 °C for 15 s, and then extension at 72 °C for 10 min. The experiment was repeated three times for each sample. The relative expression levels of the target genes were determined using the comparative CT method known as the 2^−ΔΔ^Ct method [32].

### 2.5. Statistical Analysis

The statistical analysis was carried out with reference to the previously described method [4,33,34,35]. All data were analyzed by using the Duncan analysis method of the SPSS 17.0 software (SPSS, Chicago, IL, USA) and there was significant variation and overall difference between the two treatments when ANOVA(*p*) < 0.05. Cosinor analysis and making of the curve chart by MATLAB R2023a software (Math Works, Natick, MA, USA) was done to evaluate daily rhythmicity. The statistical significance *p*-value of the cosinor analysis, which was expressed by Acro(*p*), was defined by the noise/signal of amplitude calculated from the ratio SE(A)/A. The rhythm characteristics of the genes’ expression were displayed when the two conditions were true, including both *p* < 0.3 by cosinor analysis and *p* < 0.05 by ANOVA. The correlation of messenger RNA (mRNA) expression levels between the core clock genes and fat metabolism genes was assessed by using Pearson’s correlation test (r).

## 3. Results

### 3.1. The HFD Altered Rhythmic mRNA Expression of the Core Clock Genes in Liver Tissue

**Figure 2:**
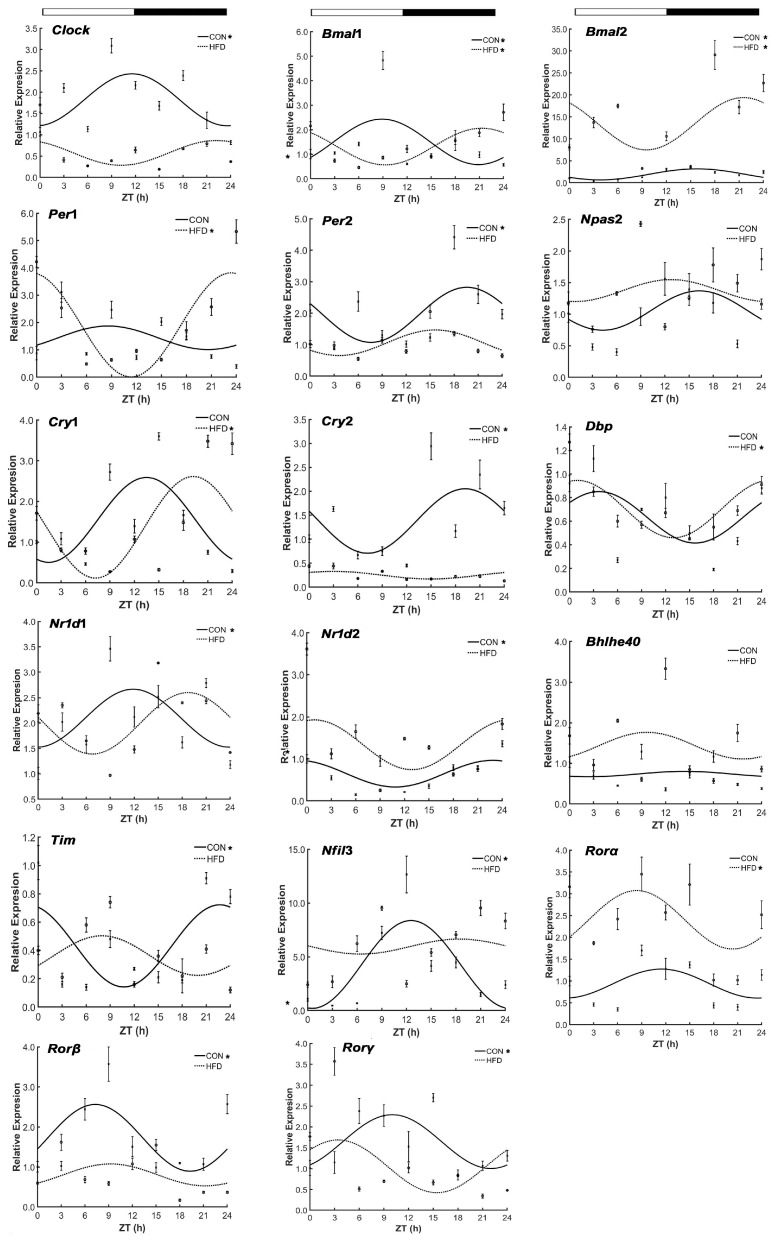
Effect of HFD on daily rhythm of the core clock genes’ expression in the liver tissue of *T. sinensis* during a daily cycle. Note: “
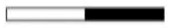
”; white part means light, black part means dark; “CON” and “HFD” represent the CON group and the HFD group, respectively; ZT indicates zeitgeber time (h); “*” shows the gene with the characteristic of circadian rhythm in the CON group and the HFD group.

The alteration influence on both circadian rhythm characteristics and mRNA expression of 17 core clock genes, *Clock*, *Bmal*1/2, *Npas*2, timeless(*Tim*), *Cry*1/2, *Per*1/2, *Dbp*, *Nfil*3, *Bhlhe*40, *Nr*1*d*1/2 and *Rorα*/*β*/*γ* of the high-fat diet (HFD) was analyzed in the liver tissues from nine turtles per group at nine time points. The results are represented in Figure 2 and Table 2. Eleven genes displayed a significant daily rhythm in the CON group (*p* < 0.05), whereas the remaining six genes, *Npas*2, *Cry*1, *Per*1, *Bhlhe*40, *Dbp* and *Rorα,* did not. Some of genes, including *Bmal*1 (ZT 8.89 h), *Rorβ* (ZT 7.31 h) and *Rorγ* (ZT 10.01 h), had peak expressions during the light phase, and others displayed daily rhythmic expression at night, such as *Bmal*2 (ZT 15.58 h), *Cry*2 (ZT 19.20 h), *Per*2 (ZT 19.60 h), *Tim* (ZT 22.69 h) and *Nr*1*d*2 (ZT 22.87 h); and three genes, *Clock* (ZT 11.57 h), *Nfil*3 (ZT 12.55 h) and *Nr*1*d*1 (ZT 11.87 h) exhibited their peaks during the alternation of the light–dark phase. However, the HFD strongly destroyed the circadian patterns of nine genes (i.e., *Clock*, *Tim*, *Cry*2, Per2, *Nfil*3, *Nr*1*d*1/2, and *Rorβ*/*γ*) through the delayed and advanced phases; however, it did not affect those of *Bmal*1 and *Bmal*2. What is more, four genes (*Cry*1, *Per*1, *Dbp* and *Rorα*) showed no diurnal rhythm in the CON group, in contrast to significant circadian rhythms in the HFD group. Of course, daily rhythmic expression was not demonstrated in either the CON group or the HFD group for the two genes *Npas*2 and *Bhlhe*40.

**Figure 3:**
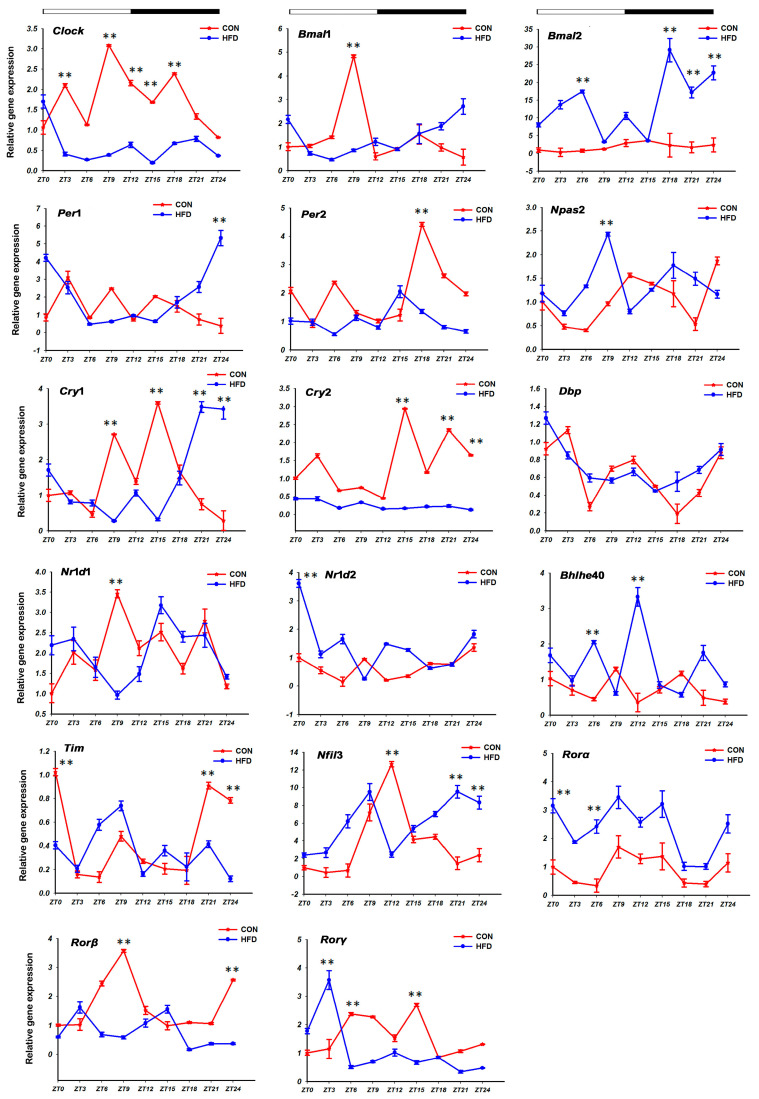
Effect of HFD on mRNA levels of the core clock genes in the liver tissue of *T. sinensis* during a daily cycle. Note: “**” shows that there was a significant difference in the gene’s mRNA levels between the CON group and the HFD group at the zeitgeber time point. The meanings of the following graphs are the same as in this note.

In addition, highly significant variations were observed for the mesors and amplitudes of 10 genes (*Clock*, *Bmal*2, *Cry*2, *Per*1/2, *Nfil*3, *Bhlhe*40, *Nr*1*d*2 and *Rorα*/*β*) between the liver tissues of the CON group and the HFD group. The results of the transcripts of the circadian clocks indicated that the mRNA expression levels of these genes were also affected by the HFD (Figure 3). Apparently, compared with the CON group, the amplitudes of the following genes in the HFD group were decreased: *Clock* (2.10-fold), *Bmal*1 (1.24-fold), *Tim* (2.07-fold), *Cry*2 (8.34-fold), *Per*2 (2.12-fold), *Nfil*3 (5.83-fold) and *Rorβ* (3.07-fold). However, they were increased for *Bmal*2, *Cry*1, *Per*1, *Nr*1*d*2 and *Rorα,* by 4.76-fold, 1.20-fold, 4.42-fold, 1.84-fold and 2.03-fold, respectively. Synchronously, three core clock gene expression levels (*Clock*, *Per*2, and *Cry*2) all had pronounced decreases in the HFD group during a 24 h cycle.

### 3.2. The HFD Altered Rhythmic mRNA Expression of the Lipid Metabolism Genes in the Liver Tissue

#### 3.2.1. The Lipid Synthesis-Related Genes

**Figure 4:**
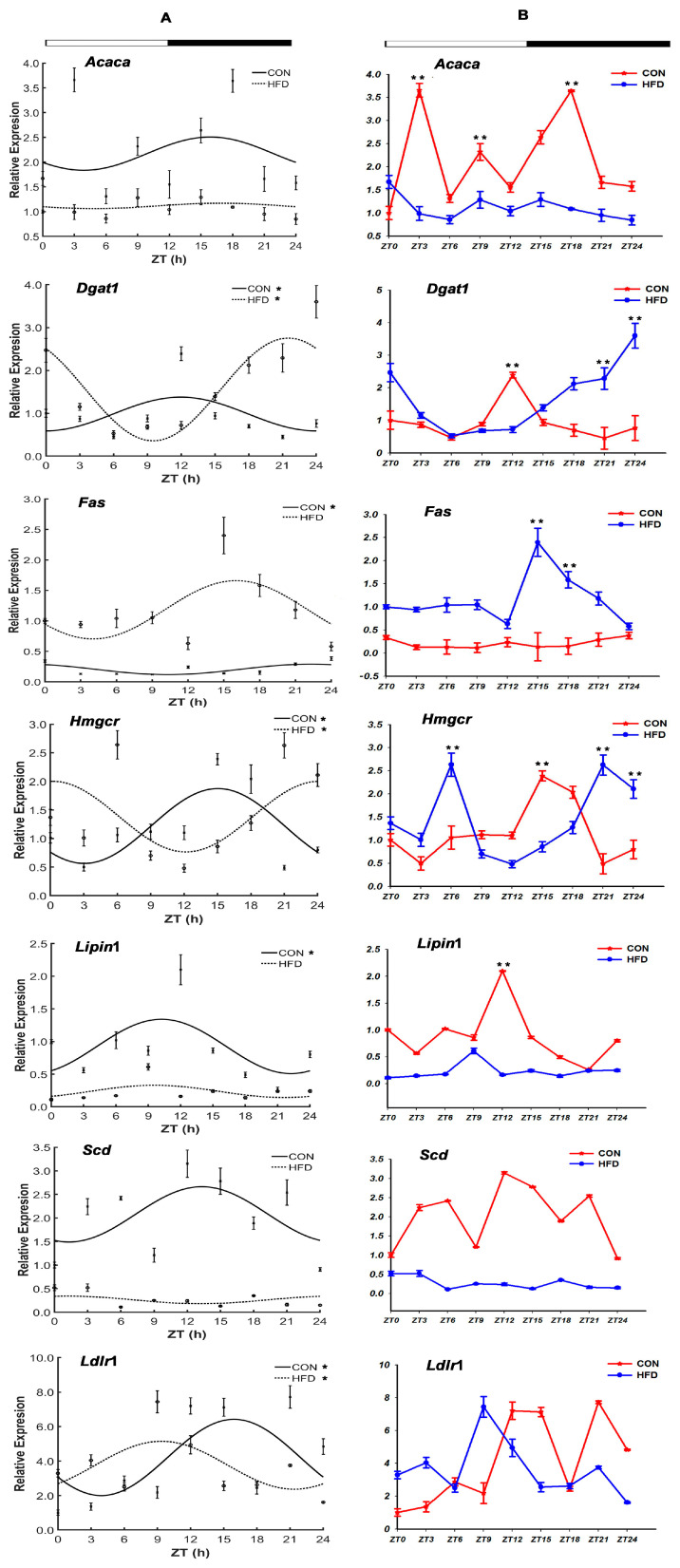
Daily rhythm and mRNA expression profiles of the lipid synthesis-related genes in the liver tissue of *T. sinensis* during a daily cycle. Note: (**A**,**B**) represent the cosine graph of daily rhythm expression and mRNA levels graph on the genes’ expression, respectively. “*” shows the gene with the characteristic of circadian rhythm in the CON group and the HFD group. “**” shows that there was a significant difference in the gene’s mRNA levels between the CON group and the HFD group at the zeitgeber time point. The meanings of the following graphs are the same as in this note.

Seven lipid synthesis-related genes were assayed in our study: fatty acid synthase (*Fas*), stearic acid dehydrogenase (*Scd*), acetoacetic acid CoA (*Acaca*), diacylglycerol acyltransferase 1 (*Dgat*1), *Hmgcr*, low-density lipoprotein receptor-related protein 1-like (*Ldlr*1), *Lipin*1 (lipin 1) (Figure 4, Table 3). In the CON group, the *Fas*, *Hmgcr* and *Ldlr*1 genes with acrophases at ZT 22.31 h, ZT 15.04 h and 15.93 h were preferentially rhythmically expressed during the dark phase; the gene of *Lipin*1 with rhythmic expression had peak acrophases at ZT 10.23 h during the light phase and the gene of *Dgat*1 with acrophases at 11.97 h presented rhythmic expression during the light on/off phase (Figure 4A). However, the genes *Acaca* and *Scd* had unexpressed circadian fashion in the two groups, and the circadian expression was also disrupted for *Fas*, *Hmgcr*, *Ldlr*1, *Lipin*1 and *Dgat*1, which showed disappeared, advanced or delayed expression peaks with the HFD. For instance, relative to the CON group, the expression peak of *Dgat*1 in the HFD group was deferred for 9.55 h, but *Lipin*1 was shifted ahead for 0.64 h. Interestingly, the expression peaks of *Hmgcr* and *Ldlr*1 genes presented a reversal of day and night, and were shifted ahead for 14.87 h and 6.56 h from the dark phase (ZT 15.04 h, ZT 15.93 h) to the light phase (ZT 0.17 h, ZT 9.37 h) (Figure 4, Table 3). Additionally, it was observed that there were highly significant variations in the mesors and amplitudes of five genes, *Fas*, *Scd*, *Acaca*, *Dgat*1 and *Lipin*1 in the two groups. 

As shown in Figure 4B, the lipid synthesis-related genes were especially significantly affected by the HFD. Compared to the CON group, there was a pronounced increase in the mRNA level of the *Fas* gene in the HFD group, whereas that of *Acaca*, *Lipin*1 and *Scd* was the opposite, and decreased over the course of a whole day (*p* < 0.05). Moreover, the levels of *Dgat*1 and *Hmgcr* showed firstly rising trends, then downward trends in the HFD group during the light phase, but presented the reverse, increasing trends, at night. And for *Ldlr*1, the mRNA level in the light phase was higher in the CON group than in the HFD group, whereas that was reversed in the dark phase. By using the highest levels of *Acaca*, *Fas*, *Hmgcr* and *Ldlr*1 in the HFD group as the reference, the mRNA levels of these genes in the HFD group corresponding with the CON group were found to be increased by 1.67-fold (ZT 0 h), 17.12-fold (ZT 15 h), 5.37-fold (ZT 21 h) and 3.41-fold (ZT 9 h), respectively.

#### 3.2.2. The Lipid Oxygenolysis-Related Genes 

**Figure 5:**
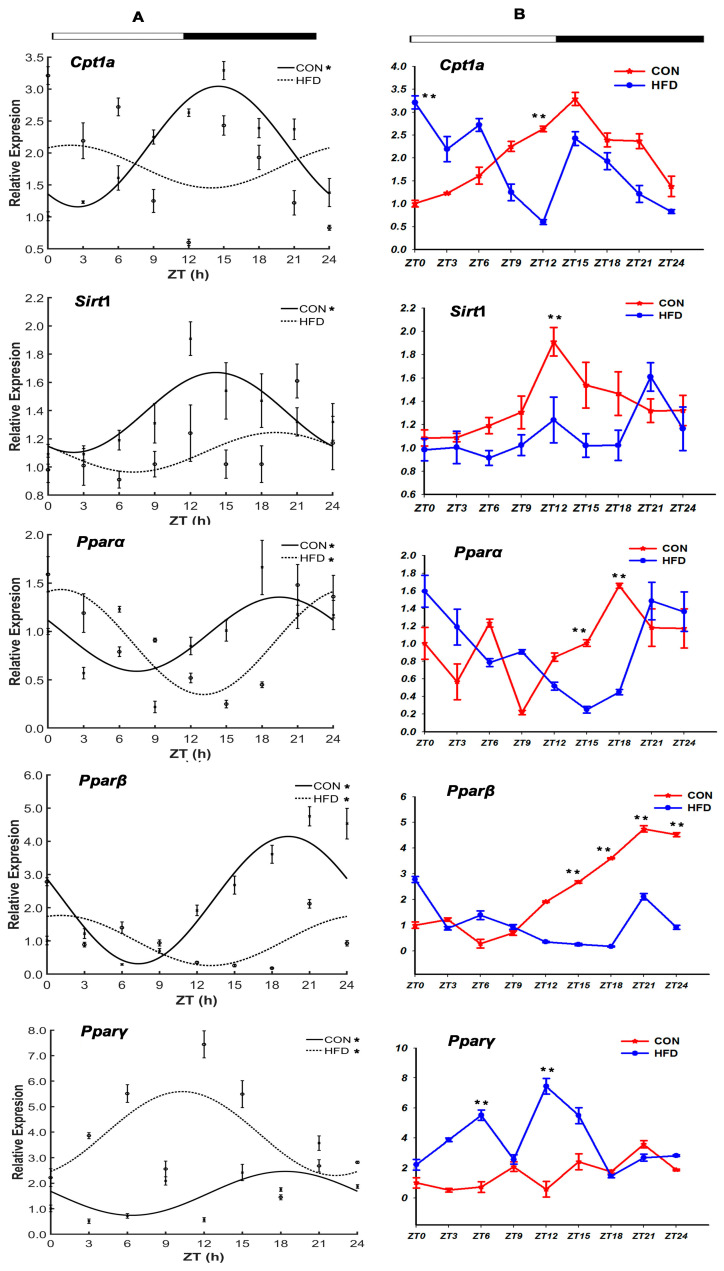
Daily rhythm and mRNA expression profiles of the lipid oxygenolysis-related genes in the liver tissue of *T. sinensis* during a daily cycle. Note: (**A**,**B**) represent the cosine graph of daily rhythm expression and mRNA levels graph on the genes’ expression, respectively. “*” shows the gene with the characteristic of circadian rhythm in the CON group and the HFD group. “**” shows that there was a significant difference in the gene’s mRNA levels between the CON group and the HFD group at the zeitgeber time point. The meanings of the following graphs are the same as in this note.

There were five genes, carnitine palmitoyltransferase 1A (*Cpt*1a), *Sirt*1, *Pparα*/*β*/*γ,* responsible for lipid oxygenolysis function (Figure 5 and Table 4). The expression peaks of the five genes all exhibited circadian expression patterns in the CON group with acrophases at ZT 14.54 h, ZT 19.47 h, ZT 19.31 h, ZT 18.36 h and 15.15 h during the dark phase. Meanwhile, those of *Cpt*1a and *Sirt*1 were disrupted in the HFD group, and the remaining three genes displayed advanced expression peaks, influenced by the HFD. To our surprise, there was a synchronization phenomenon, not only for the circadian expression patterns of *Pparα* (ZT 1.07 h) and *Pparβ* (ZT 1.07 h), but additionally, their expression peaks were shifted ahead for almost 18 h in unison from the dark phase to the light phase in the CON group. Similarly, the expression peak of *Pparγ* was also shifted ahead for 12.62 h from the dark phase to the light phase in the CON group, in reverse. Compared with the CON group, the mRNA expressions of the five genes in the HFD group were significantly altered during the dark and light phases (*p* < 0.05). As Figure 5B shows, the mRNA levels of *Pparγ*, which was opposite to *Sirt*1, were observably higher in the HFD group than in the CON group (*p* < 0.05). In addition, compared with the CON group, the *Pparα* mRNA levels showed firstly a rising trend, then a downward trend, and lastly an increasing trend in the HFD group. Differently, the mRNA levels of *Cpt*1a and *Pparβ* in the HFD group had firstly rising trends, then decreasing trends (*p* < 0.05). 

#### 3.2.3. The Lipid Transport-Related Genes 

**Figure 6:**
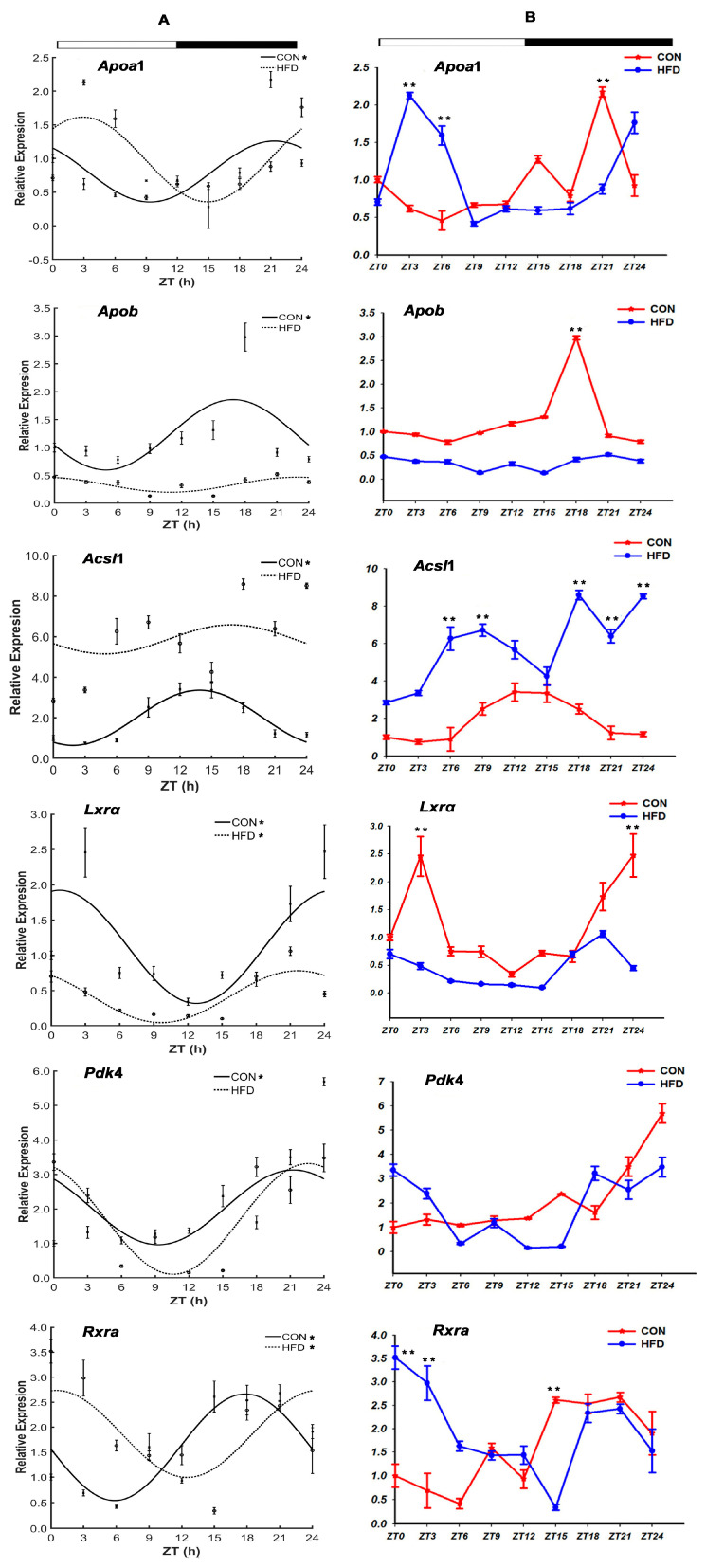
Daily rhythm and mRNA expression profiles of the lipid transport-related genes in the liver tissue of *T. sinensis* during a daily cycle. Note: (**A**,**B**) represent the cosine graph of daily rhythm expression and mRNA levels graph on the genes’ expression, respectively. “*” shows the gene with the characteristic of circadian rhythm in the CON group and the HFD group. “**” shows that there was a significant difference in the gene’s mRNA levels between the CON group and the HFD group at the zeitgeber time point. The meanings of the following graphs are the same as in this note.

The present results revealed that the six lipid transport-related genes all exhibited circadian oscillation in the CON group, which contained apolipoprotein A1 (*Apoa*1), apolipoprotein B (*Apob*), acyl-CoA synthase long-chain1 (*Acsl*1), liver X receptor α (*Lxrα*), pyruvate Dehydrogenase kinase 4 (*Pdk*4) and retinoid X receptor α (*Rxra*) (Figure 6, Table 5). However, it was significantly altered for these genes by the administration of a high-fat diet. For instance, the diurnal rhythms of four genes, *Apoa*1, *Apob*, *Pdk*4 and *Acsl*1, were dampened by the HFD, and the mRNA peaks of *Rxra* and *Lxrα* were shifted ahead by 17.36 h (*Rxra*, ZT 0.49 h) and postponed by 20.92 h (*Lxrα*, ZT 21.66 h), while still being held for the circadian rhythm in the HFD group. Furthermore, compared with the CON group, the mRNA levels of *Acsl*1 showed an evidently rising trend, and those of *Apob* and *Lxrα* had contrary decreasing trends in the HFD group (*p* < 0.05), while those of the three genes *Apoa*1, *Pdk*4 and *Rxra* had firstly rising trends, then decreasing trends (*p* < 0.05). 

### 3.3. The Correlation Analysis on Daily Expression between the Core Clock Genes and the Lipid Metabolism Genes in Liver Tissue

In the present study, there were either positive or negative correlations for the daily expression levels between the core clock genes and the lipid metabolism genes in liver tissues (Table 6 and Table 7). There were different results for the correlation relationships between the CON group and the HFD group. Of these core clock genes, the transcription level of *Clock* presented a strong positive correlation with the daily expression of *Bmal*1 (r = 0.71) and moderate positive correlations with the daily expressions of *Per*2, *Nr*1*d*1 and *Nfil*3 (r = 0.51, 0.63 and 0.56) in the CON group. Here, *Bmal*1 was also strongly positively correlated with *Tim* (r = 0.81), and had a moderate positive correlation with *Nr*1*d*1 (r = 0.63), while *Bmal*2 showed moderate positive correlations with *Per*2 and *Nfil*3 (r = 0.50 and 0.54). In addition, *Per*2 and *Cry*2, *Nr*1*d*2 and *Tim* both had moderate positive correlations with each other (0.5 ≤ r < 0.7). However, many pairs of correlation genes in the CON group disappeared in the HFD group. Particularly, there were only strong positive correlation pairs within components of the genes *Bmal*1: *Cry*1/ *Per*1 (r = 0.84 and 0.81), and moderate positive correlation pairs like *Bmal*1: *Dbp* (r = 0.58), and its sibling gene *Bmal*2 had a strong negative correlation with *Rorα* (r = −0.75). In addition, *Per*1 displayed strong positive correlations with the two genes *Cry*1 and *Dbp* (r = 0.74 and 0.79) in the HFD group.

In the meantime, we found that there were positive/negative correlations of the transcription levels between the core clock genes and the lipid metabolism genes in the CON group. Firstly, many pairs of transcription levels exhibited moderate or strong positive correlation relationships as follows: *Bmal*2: *Ldlr*1/*Hmgcr*, *Cry*2: *Fas*, *Per*2: *Hmgcr*/*Fas*, *Nfil*3: *Dgat*1 (r ≥ 0.50). Meanwhile, the pair *Cry*2: *Lipin*1 showed a negative correlation (r = −0.54). However, in the HFD group, the correlation relationships were altered among these pairs. For example, there were correlation relationships between only the three lipid synthesis genes *Dgat*1, *Hmgcr* and *Ldlr*1 and the core clock genes. Furthermore, *Dgat*1 was positively administrated by *Bmal*1, *Per*1, *Cry*1 and *Dbp* (r ≥ 0.50), and *Hmgcr* also had positive relationships with the other core clock genes *Cry*1 and *Bmal*2 (r ≥ 0.50), while *Ldlr*1 was negatively regulated by *Bmal*2 (r = −0.56). Furthermore, there were also strong positive correlation relationships among these pairs, *Bmal*2: *Cpt*1a, *Bmal*2/*Nfil*3: *Sirt*1 (r ≥ 0.7) and moderately positive correlation relationships among the pairs *Per*2/*Nr*1*d*1/*Nfil*3: *Cpt*1a, *Bmal*2/*Cry*2: *Pparβ*, *Cry*2: *Pparγ*, *Nr*1*d*2/*Clock*: *Lxrα* and *Bmal*1: *Pparα* (0.5 ≤ r < 0.7) in the CON group. However, the correlation relationships of the above pairs were seriously disturbed by the HFD. In the HFD group, the pairs were replaced with the following correlation relationships: *Dbp*/*Per*1/*Cry*1/ *Bmal*1: *Pparα* (r = 0.77, 0.73, 0.62 and 0.53), *Per*1: *Pparγ* (r = 0.50), *Bmal*2: *Pparβ* (r = −0.52). Furthermore, the positive correlation relationships were shown in both the core clock genes (i.e., *Bmal*2, *Clock*, *Per*2, *Cry*2, *Nr*1*d*2, *Tim*) and the lipid metabolism genes (i.e., *Acsl*1, *Rxra*, *Apoa*1) (r ≥ 0.50), while those core clock genes that contained *Clock*, *Bmal*1/2, *Cry*1, *Dbp* and *Per*1 maintained positive/negative correlation relationships together with the three transport-related genes including *Lxrα* and *Rxra*.

## 4. Discussion

Some studies have indicated that diseases like obesity, metabolic syndrome and even diabetes have come about due to daily rhythm alterations and expression disorders of the core clock genes [36]. Further research has shown that by feeding them a high-fat diet (HFD), it is possible to alter mice’s daily rhythm and energy metabolism systems, resulting in the occurrence of metabolic diseases. These studies were mainly focused on mammals, but little attention has been given to aquatic animals [37,38,39]. In the present study, we aimed to explore the influence of circadian rhythm features and mRNA levels on the core clock genes and the lipid metabolism genes, together with correlativity for these genes with a high-fat diet, in the liver tissues of *T. sinensis*. 

It had already been verified that high-fat diets could alter circadian rhythms and affect metabolic physiology in both brain and peripheral tissues [4,23]. In recent years, the eating of a high-fat diet—representing an unhealthy lifestyle—has led to a rise in obesity groups, whose liver hormones and hormone receptors are involved in the core clock genes’ being delayed or altered, together with changes in the genes’ expression from the adiponectin signal components [40]. In the present study, we observed similar results; 11 genes (*Clock*, *Bmal*1/2, *Tim*, *Cry*2, *Per*2, *Nfil*3, *Nr*1*d*1/2 and *Rorβ*/*γ*) revealed circadian oscillation by cosine analysis. This was consistent with the previous research in that the disappearance of daily rhythms was displayed for the genes *Clock*, *Cry*2, *Per*2, *Nfil*3, *Nr*1*d*1/2 and *Rorβ*/*γ*, and peak phase changes were presented in three genes, *Bmal*1/2 and *Tim*, and the difference in amplitudes, with their mRNA levels, were also shown in the CON and HFD groups [41,42]. What is more, in this study, *Cry*1, *Per*1, *Dbp* and *Rorα* in the HFD group had circadian oscillation emergence, instead of being shown to have no diurnal rhythm, as in the CON group. Research has also found that the rhythmic expression patterns of the core clock genes including *Clock*, *Bmal*1, *Per*2 and *Cry*2 in mice were changed by eating a high-fat diet, while the two genes *Per*1 and *Cry*1 presented well circadian oscillation characteristics in either a CON group or in a HFD group [41,42]. The result indicates the conclusion that the two genes played roles in mice’s fatty liver disease caused by a high-fat diet. This was different from the present study; we predicted that the circadian rhythms system was not absolutely regulated by *Per*1 and *Cry*1, but was run by the other genes in turtle liver tissue. Some reports showed that a high-fat diet had little effect on the expression of circadian rhythm genes in C57BL/6 female mice [43,44]. Kohsaka and his colleagues explored the way in which the mRNA levels of the core clock genes were significantly changed after feeding C57BL/6 male mice a high-fat diet for 6 weeks [19]. Consistent with the previous study, high-fat feeding reduced the amplitude of the locomotor activity rhythm in male C57BL/6J mice and did not alter the amplitude of the locomotor activity rhythm in female mice [45,46]. A possible explanation for the differences might be differences in the animal species, their growth stages, the feeding times of the HFD or tissue samples [17]. Thus, we derived the opinion through the study that the animals could bear the expression changes of these core clock genes, resulting in their making corresponding physiological adjustments, which were accordingly suitable for changes in the external environment (such as food), so as to more quickly and better adapt to the new environment, under suitable conditions. This ability would be completely destroyed, resulting in suffering from occurrences of obesity, metabolic syndrome and other diseases, if this clock system was beyond the scope of their own ability [47]. Therefore, the fat content of the feed should not be so high as to to exceed the environmental tolerance of *T. sinensis*. 

The research has reported that the role of the circadian clock was involved in the regulation of lipid metabolism in teleost fish [48]. It was also verified that an HFD could disrupt the mechanism of the biological clock system, and destroy the rhythmic expression of metabolic enzyme genes, which were affected by the physiological mechanism in *Bmal*1 together with *Nr*1*d*1 that regulates adipocyte differentiation [43]. Moreover, hypertriglyceridemia came from *Clock* gene mutations to induce disturbances in the rhythm expression of *Bmal*1 and *Per*2 genes, and thus led to the rhythm expression’s disappearance in *Ldlr* and *Hmgcr* genes, which run lipid synthesis in mice livers [36]. The functions of cholesterol synthesis and oxygenolysis were controlled by the circadian rhythm expression of genes including *Hmgcr*, *Ldlr*1, *Apob* and *Apoe* in live subjects [16]. In our study, we found that three genes, *Fas*, *Scd* and *Lipin*l, had circadian oscillation in the CON group, instead of non-circadian rhythms in the HFD group, and the mRNA peak phases of *Hmgcr* and *Ldlr1* genes were advanced and delayed. A high-fat diet remarkably decreased the mRNA levels of the mouse *Hmgcr* gene at the ZT 8 h point, but did not destroy its circadian oscillation, while it delayed the expression peak of the *Ldlr*1 gene (for about 6 h). Meanwhile, the mRNA expression peak of *Dgat*1 was reversed from the light day to the dark day, and the circadian oscillation of *Fas* was broken with significant improvement in its mRNA level. The result showed that it was coupled with an increase in triglycerides concentration, implying that these genes may become the potential genes for triglyceride accumulation in the liver by high-fat-diet induction [41]. Insulin levels continued to rise, because of them being fed an HFD, in mice serum, synchronously causing a decline in expression levels and a disappearance of circadian rhythms for *Bmal*1 and *Fas* genes [49]. It was reported that *Lipin*l knockout mice showed changes in their core clock genes’ expression, like *Bmal*l and *Clock* [50]. In the CON group of the study, the *Fas* gene was strongly positively correlated with *Cry*2 and *Per*2 genes, which were located in the negative feedback loop, while *Hmgcr* was also strongly correlated with both *Bmal*2 and *Per*2, which were located in the positive and negative feedback loops, respectively. And the result was in accord with the research, as Turek et al. reported [51]. However, in the HFD group, results were different in that *Hmgcr* and *Dgat*1 had strong relationships with the core clock genes including *Bmal*1/2, *Cry*1, *Per*1 and *Dbp*, which implied that the high-fat diet affected the expression of the core clock genes and the lipid synthesis-related genes. 

In the present study, we also explored the idea that *Pparα* mRNA expression had a decreasing trend from ZT 12 h to ZT 21 h; both *Clock* and *Cry*2 mRNA expression in the HFD group presented remarkably lower than that in the CON group at 24 h. The report indicated that *Pparα* mRNA expression also remarkably decreased at ZT 12 h, ZT 18 h and ZT 24 h, and the mRNA levels of *Clock* and *Bmal*1 genes had the same trends in the night time in the livers of the HFD mice. The differences between *T. sinensis* and the mice might be due to the variations in animal species [52,53]. Research showed that the rhythm expression character of the *Pparα* gene in liver tissue was interdependent with the rhythm expression of *Clock* and *Bmal*1, and the *Pparα* and *Pparβ* expression levels in liver and intestinal tissues were decreased as dietary lipids increased from 5% to 11%, while being up-regulated in fish being fed choline supplementation of 1800 mg kg^−1^ [54,55]. Similarly, when the expression of *Per*2 was suppressed or knocked out, the *Pparγ* of mice was activated to provoke abnormal fat metabolism, while *Pparγ’s* absence led to a loss of circadian rhythm expression of genes such as *Clock*, *Bmal*1, *Per* and *Cry* [56]. Moreover, the two genes *Cpt*1a and *Sirt*1 played important roles in lipolysis metabolism and β oxidation, which were regulated by the core clock genes including *Bmal*1, *Cry*1, *Rorα* and *Per*2 [57]. In our study, it was also shown that the circadian rhythm expressions of *Cpt*1a, *Sirt*1 and *Ppars* were lost or changed due to the significant changes in the expression of the core genes. Furthermore, the correlated relationships of all these genes were influenced by an HFD in the liver tissues of *T. sinensis*. For instance, the strong positive correlations for *Cpt*1a vs. *Bmal*2, Stirt vs. *Bmal*2, together with the positive correlations for *Pparβ* vs. *Bmal*2, *Pparγ* vs. *Cry*2, *Pparα* vs. *Bmal*1 in the CON group were broken in the HFD group. These results convincingly supported that the expressions of the core circadian clock genes were affected by the HFD, which led to the expression changes related to the lipid metabolism genes in the liver tissues of *T. sinensis*.

It is generally acknowledged that the lipid transport function works as a link and a balance between both lipid synthesis and lipolysis oxidation. It is operated by the lipid transport genes and their transcription factors and decides the amount of fat to be deposited in the body. The genes *Acsl*1 (long-chain acyl coenzyme A synthase), the five members of the apolipoprotein family (AopA, AopB, AopC, AopD and AopE) and three other genes, *Fas*, *Scd* and *Lxrα,* have characteristic rhythmic expressions, and are responsible for the transport of fatty acids and cholesterol in animals [57]. Particularly, among these members, the expressions of *Apoa*1, *Apob*, *Fas*, *Scd* and *Lxrα* genes were shown to be low in the daytime and high in the evening because of the key role of the *Clock* gene in mice in this process [58]. It was surprising to discover that there were strong positive correlations for the mRNA levels of *Pparα* together with the three genes *Apoa*1, *Apob* and *Pdk*4 [59]. Thus, it was concluded that a physiological function of the body was regulated not by a single gene, but was co-worked by multiple genes and pathways. For example, the two genes *Pparα* and *Rxra* were dimerized and interacted together to activate the CLOCK protein ligand, and further inhibit the CLOCK/ BMAL1 protein activity via its E-box element [60]. Similarly, the result in our study showed that the four lipid transporter genes of the circadian expression (*Apoa*1, *Apob*, *Pdk*4, *Acsl*1) in the CON group were altered or faded away, and the rhythm phase of two genes (*Lxrα* and *Rxra*) were shifted, whose mRNA levels were changed correspondingly by an HFD. Simultaneously, the presented positive or moderate correlation relationships for genes including *Per*2, *Clock*, *Bmal*1, *Nr*1*d*1 and *Lxrα* in the CON group were all replaced by the positive or moderate correlation relationships of the three genes *Cry*1, *Bmal*1 and *Lxrα* in the HFD group. So, it was speculated that the expression levels of the core circadian genes in the liver tissues of *T. sinensis* were altered by the high-fat diet, mating the changes in the mRNA levels of the fat transporter genes and the transcriptional genes. However, a high-fat diet could alter the daily rhythm and the strong correlation between the transcription levels of the core clock genes and the lipid metabolism genes. There is plausible evidence that synchronized rhythmic oscillations were altered by the core clock genes through the regulation of the lipid metabolism genes in response to the high-fat diet. In sum, it is not advisable to reduce the amount of protein by increasing the fat content in the feed so as to reduce the feed cost. In fact, the result would not only disorder the physiological rhythm of *T. sinensis*, but also cause the disease of fatty liver, resulting in it being bad for the health of both *T. sinensis* and the consumers who like to eat Chinese soft-shelled turtles.

## 5. Conclusions

In the present study, we first investigated the influence of a high-fat diet on the core clock genes and the lipid metabolism gene expressions in the liver tissues of *T. sinensis*. Our results showed that the core circadian genes were in charge of regulating the expression of lipid metabolism genes and maintaining their physiological activity, which were all disturbed by the high-fat diet. To sum up, the laws of the life activities of *T. sinensis* were revealed by a circadian rhythm mechanism and their mutual interaction. The result supports a new perspective to guide the production practices of the aquaculture industry.

## Figures and Tables

**Table 1:** Primers used for the genes’ qRT-PCR in the liver tissue of *T. sinensis.*

Gene Name	Forward and Reverse Primers Sequence (5′-3′)	Annealing Temperature (°C)	Product Sizes (bp)	GeneBank Accession No.
*Clock*	F 5′ GTCATCGCTTAGTAGTCAGTCCTT 3′	57.0	187	XM_014568700.2
	R 5′ TATCATTCGTGTTCTTTGCTCC 3′	57.3		
*Bmal*1	F 5′ GATAAAGATGACCAACACGGAAGG 3′	61.9	338	XM_014568878.2
	R 5′ TCACAGCCCACAACAAACAGAA 3′	61.3		
*Bmal*2	F 5′ ACATTACTACCCTGTGGTTCCC 3′	57.4	287	XM_006127687.3
	R 5′ GTCTCCAAGTCCTCCATTTCTG 3′	57.9		
*Npas*2	F 5′ AGGCATTAGATGGCTTCGTTAT 3′	58.0	145	XM_014578632.2
	R 5′ GAATGTTCTTGTTCTGGGAGGA 3′	58.3		
*Tim*	F 5′ TGGGAGCAGAGGCAGGAG 3′	58.8	248	XM_025188709.1
	R 5′ CTGAACATGAGCGAGACGATTT 3′	59.6		
*Cry*1	F 5′ GTTGGATTCACCACCTTGCTC 3′	59.3	300	XM_025178935.1
	R 5′ GTGCTGTCCAAGGCTCGTAG 3′	58.2		
*Cry*2	F 5′ CTGTTTATTGGCATCAGTCCCT 3′	58.6	154	XM_006124501.2
	R 5′ CTCCTCTATTCCCTCATGTTTACG 3′	59.5		
*Per*1	F 5′ TGCGTCAAGCAGGTCCAAG 3′	60.1	167	XM_025181282.1
	R 5′ GAGACAGCCACGGCAAAGG 3′	61.2		
*Per*2	F 5′ CACCTTCTTGTCCCTCTATCCA 3′	58.4	234	XM_042853554.1
	R 5′ TCTTTGCCCACGAGTACCATG 3′	60.9		
*Dbp*	F 5′ ATGAACTTTGACCCTGACCCTG 3′	60.5	136	XM_006132772.2
	R 5′ GGATTTTCCGTGCCTTCTTCAT 3′	62.3		
*Nfil*3	F 5′ TCTGTGGTGGGCAGTAGTTGTA 3′	58.2	291	XM_006131880.3
	R 5′ ATTCACTTGTAGCAGAGGAGGG 3′	57.9		
*Bhlhe*40	F 5′ ACAGACAGTGGGTATGGAGGAG 3′	58.0	294	XM_006132802.3
	R 5′ CAGCATAGGCAGATAGGCAGTT 3′	59.2		
*Nr*1*d*1	F 5′ TCCTGAGCGGCGAGACCTAC 3′	62.5	256	XM_025179480.1
	R 5′ GAGTCATCGGGGTGCTTCTTT 3′	60.7		
*Nr*1*d*2	F 5′ CAATGGCTACCAGGGCAACA 3′	61.6	347	XM_014579147.2
	R 5′ GCTTGGCAAACTCCACTACCTC 3′	60.7		
*Ror* *α*	F 5′ CATCGGGCTTCTTCCCTTATT 3′	60.2	207	XM_025178325.1
	R 5′ TTACCTCCCTCTGCTTGTTCTG 3′	58.9		
*Ror* *β*	F 5′ CTGCAAGGGTTTCTTTAGGAGG 3′	60.2	338	XM_006137819.3
	R 5′ AGTAAGTGCCACCAGTTTCGTT 3′	58.4		
*Ror* *γ*	F 5′ CTACACCAGTCCCAACTTCACCA 3′	61.5	208	XM_025182756.1
	R 5′ CCGTTCCCACATCTCCTCCA 3′	62.7		
*Acaca*	F 5′ CGTCCGAGAACCCCAAACTA 3′	60.0	264	XM_006138625.3
	R 5′ CCAGCAACCCATCATCCAC 3′	58.8		
*Dgat*1	F 5′ TTGCTGCCTCTGTTTTGTTTG 3′	59.1	167	XM_014576010.1
	R 5′ TGACTGTCCTCTTTCGTTCCTTC 3′	60.2		
*Fas*	F 5′ CGTGGGCTTGGCTGCTATTC 3′	63.0	249	XM_014580710.1
	R 5′ GGAGGACAACGGCTCTTACATT 3′	60.1		
*Hmgcr*	F 5′ TCATCAGTCTCGCTGGTCGTA 3′	58.9	235	XM_014574160.1
	R 5′ GGAATGACTGCTTCACAGACCA 3′	59.9		
*Lipin*1	F 5′ CACTGGGTGAACGAACGAGG 3′	61.0	191	XM_006138555.2
	R 5′ GCAGGTCTGTTTCCAAAGGCT 3′	60.9		
*Scd*	F 5′ GAGGTTTTACAAGCCTTCCGTG 3′	60.8	291	XM_014579917.1
	R 5′ TCGCTGGTGGCGTAGTCGT 3′	62.1		
*Ldlr*1	F 5′ CCAACGCTCAGCAGAAAACC 3′	60.7	166	XM_014577039.1
	R 5′ GGTTTGCCGAACTGGTCTTG 3′	60.4		
*Cpt*1a	F 5′ GAGCAGGGATACAGGGAAGAGG 3′	61.8	193	XM_006131643.1
	R 5′ CATTCTCCCAAAGGTGTCCAAC 3′	60.9		
*Sirt*1	F 5′ AGTAGACTTCCCAGACCTTCCAG 3′	58.6	208	XM_006125276.3
	R 5′ AACCTGTTCCAGCGTATCTATGT 3′	57.7		
*Pparα*	F 5′ AGAGGAGGATGATCTCAGAAACC 3′	58.3	186	XM_014575807.1
	R 5′ GATGCTGGTGAAAGGGTGTCTG 3′	60.8		
*Pparβ*	F 5′ GGAGGACCAGACCGTTTGCC 3′	63.8	167	XM_006115662.2
	R 5′ CCGTAATGAAATCCCGATGCTA 3′	61.8		
*Pparγ*	F 5′ GTGGAGACAAGGCTTCTGGATT 3′	59.9	216	XM_006117601.2
	R 5′ GCATTCGCCCAAACCTGATA 3′	60.9		
*Rxra*	F 5′ AAGGACCGAAATGAGAACGAGG 3′	62.3	221	XM_014580328.1
	R 5′ ATTCGCTTTGCCCATTCCAC 3′	62.3		
*Lxrα*	F 5′ AGACCCTCATAACCGTGAAGCA 3′	61.2	215	XM_006135352.2
	R 5′ TGATGCTTTCAGTCTCTGGATTGTA 3′	61.0		
*Pdk*4	F 5′ CAGTCCGAAATAGACACCACGAT 3′	60.8	253	XM_014569249.2
	R 5′ TTCACCACTCCCACCACATCAC 3′	62.5		
*Acsl*1	F 5′ ATACAGGCAAGTCTGGGAGGAA 3′	60.4	128	XM_006128490.2
	R 5′ TCAGTTTGTCCATAGCCTTCGT 3′	59.1		
*Apoa*1	F 5′ GCTGGCTCCCTACTACACGC 3′	59.9	223	XM_006121675.2
	R 5′ CAGGACCTCCATCTTCTGCTTG 3′	61.3		
*Apob*	F 5′ GACTGAACAGCCCATTAGCCA 3′	60.1	160	XM_014577946.2
	R 5′ GTGACTTGTGCCATCATACCGT 3′	59.8		
*Rpl*19	F 5′ TCGTATGCCCGAGAAGGTGA 3′	61.2	180	XM_006126213.1
	R 5′ GCCTTGAGTTTGTGGATGTGCT 3′	61.6		

**Table 2:** Rhythmicity parameters of the core clock genes’ transcriptions in the liver tissue of *T. sinensis.*

Gene Name	Mesor		Amplitude		Acro(*p*)		Peak of Expression/ZT(h)		ANOVA(*p*)	
	CON	HFD	CON	HFD	CON	HFD	CON	HFD	CON	HFD
***Clock***	1.82	0.57	0.61	0.29	**0.12**	0.34	11.57	22.20	**<0.05**	<0.05
***Bmal*** **1**	1.51	1.31	0.93	0.75	**0.28**	**0.03**	8.89	21.30	**<0.05**	**<0.05**
***Bmal*** **2**	1.91	13.43	1.25	5.95	**0.01**	**0.28**	15.80	21.53	**<0.05**	**<0.05**
*Npas*2	1.06	1.37	0.31	0.17	0.42	0.75	16.26	12.77	<0.05	<0.05
***Tim***	0.43	0.36	0.29	0.14	**0.12**	0.33	22.69	7.96	**<0.05**	<0.05
***Cry*** **1**	1.55	1.36	1.04	1.25	0.04	**0.02**	13.49	21.89	n.s.	**<0.05**
***Cry*** **2**	1.38	0.25	0.67	0.08	**0.19**	0.33	19.20	2.97	**<0.05**	n.s.
***Per*** **1**	1.44	1.91	0.43	1.90	0.61	**0.01**	8.59	23.36	<0.05	**<0.05**
***Per*** **2**	1.95	1.06	0.87	0.40	**0.20**	0.11	19.60	15.62	**<0.05**	n.s.
***Dbp***	0.63	0.70	0.22	0.24	0.31	**0.03**	3.68	0.99	<0.05	**<0.05**
***Nfil*** **3**	4.29	5.95	4.08	0.70	**0.02**	0.89	12.55	18.42	**<0.05**	<0.05
*Bhlhe*40	0.74	1.44	0.07	0.33	0.92	0.73	14.40	9.67	<0.05	<0.05
***Nr1d*** **1**	2.10	2.00	0.57	0.61	**0.22**	0.13	11.87	18.75	**<0.05**	n.s.
***Nr1d*** **2**	0.65	1.34	0.32	0.59	**0.15**	0.36	22.87	0.90	**<0.05**	<0.05
***Ror*** ***α***	0.94	2.40	0.33	0.67	0.29	**0.24**	11.46	8.39	n.s.	**<0.05**
***Ror*** ***β***	1.73	0.80	0.83	0.27	**0.13**	0.51	7.31	9.23	**<0.05**	<0.05
***Ror*** ***γ***	1.65	1.06	0.64	0.63	**0.05**	0.39	10.01	3.43	**<0.05**	<0.05

Note: The bold font indicates that the gene had rhythmical characteristics, and n.s meant insignificant difference.

**Table 3:** Rhythmicity parameters of the lipid synthesis-related genes’ transcription in the liver tissue of *T. sinensis.*

Gene Name	Mesor		Amplitude		Acro(*p*)		Peak of Expression/ZT(h)		ANOVA(*p*)	
	CON	HFD	CON	HFD	CON	HFD	CON	HFD	CON	HFD
*Acaca*	2.17	1.12	0.34	0.05	0.77	0.91	15.88	16.86	<0.05	<0.05
***Dgat*** **1**	0.98	1.56	0.39	1.20	**0.26**	**0.00**	11.97	21.52	**<0.05**	**<0.05**
***Fas***	0.20	1.18	0.09	0.48	**0.11**	0.13	22.31	16.04	**<0.05**	n.s.
***Hmgcr***	1.22	1.38	0.65	0.62	**0.03**	**0.18**	15.04	0.17	**<0.05**	**<0.05**
***Lipin*1**	0.92	0.24	0.42	0.09	**0.15**	0.37	10.23	9.59	**<0.05**	<0.05
*Scd*	2.08	0.26	0.59	0.08	0.22	0.52	13.32	1.21	n.s.	n.s.
***Ldlr*** **1**	4.21	3.76	2.21	1.39	**0.17**	**0.16**	15.93	9.37	**<0.05**	**<0.05**

Note: The bold font indicates that the gene had rhythmical characteristics, and n.s meant insignificant difference.

**Table 4:** Rhythmicity parameters of the lipid oxygenolysis-related genes’ transcription in the liver tissue of *T. sinensis.*

Gene Name	Mesor		Amplitude		Acro(*p*)		Peak of Expression/ZT(h)		ANOVA(*p*)	
	CON	HFD	CON	HFD	CON	HFD	CON	HFD	CON	HFD
***Cpt*1a**	2.10	1.79	0.94	0.33	**0.01**	0.71	14.54	1.92	**<0.05**	<0.05
***Sirt*1**	0.93	1.24	0.28	0.41	**0.21**	0.23	23.46	1.29	**<0.05**	n.s.
***Pparα***	0.97	0.89	0.38	0.54	**0.10**	**0.02**	19.47	1.07	**<0.05**	**<0.05**
***Pparβ***	2.23	1.01	1.92	0.75	**0.01**	**0.09**	19.31	1.07	**<0.05**	**<0.05**
***Pparγ***	1.60	3.95	0.86	1.64	**0.17**	**0.12**	18.36	10.30	**<0.05**	**<0.05**

Note: The bold font indicates that the gene had rhythmical characteristics, and n.s meant insignificant difference.

**Table 5:** Rhythmicity parameters of the lipid transport-related genes in the liver tissue of *T. sinensis*.

Gene Name	Mesor		Amplitude		Acro(*p*)		Peak of Expression/ZT(h)		ANOVA(*p*)	
	CON	HFD	CON	HFD	CON	HFD	CON	HFD	CON	HFD
***Apoa*** **1**	0.81	0.98	0.45	0.63	**0.14**	0.03	21.37	2.90	**<0.05**	n.s.
***Apob***	1.23	0.33	0.63	0.14	**0.11**	0.03	16.87	22.94	**<0.05**	n.s.
***Acsl*** **1**	2.00	5.87	1.36	0.71	**0.00**	0.77	13.86	16.88	**<0.05**	<0.05
***Lxrα***	1.27	0.41	0.65	0.37	**0.15**	**0.01**	14.06	21.66	**<0.05**	**<0.05**
***Pdk*** **4**	4.29	5.95	4.08	0.70	**0.02**	0.89	12.55	18.42	**<0.05**	<0.05
*Rxra*	1.60	1.86	1.06	0.87	**0.01**	**0.06**	17.85	0.49	**<0.05**	**<0.05**

Note: The bold font indicates that the gene had rhythmical characteristics, and n.s meant insignificant difference.

**Table 6:** Correlation indices between the core clock genes in the liver tissue of the CON group.

Gene	Gene	Correlation Coefficient r	Gene	Gene	Correlation Coefficient r	Gene	Gene	Correlation Coefficient r
*Clock*	*Bmal*1	0.71	*Clock*	*Per*2	0.51	*Bmal*2	*Nfil*3	0.54
*Clock*	*Nr*1*d*1	0.63	*Nr*1*d*2	*Tim*	0.69	*Bmal*2	*Per*2	0.50
*Clock*	*Nfil*3	0.56	*Bmal*1	*Tim*	0.81	*Per*2	*Cry*2	0.54
*Bmal*1	*Nr*1*d*1	0.63	*Nfil*3	*Dgat*1	0.84	*Bmal*2	*Hmgcr*	0.64
*Per*2	*Fas*	0.84	*Bmal*2	*Ldlr*1	0.72	*Per*2	*Hmgcr*	0.80
*Cry*2	*Fas*	0.61	*Cry*2	*Pparβ*	0.55	*Nfil*3	*Sirt*1	0.80
*Nfil*3	*Cpt*1a	0.56	*Bmal*2	*Pparβ*	0.51	*Bmal*2	Stirt	0.72
*Per*2	*Cpt*1a	0.63	*Tim*	*Apoa*1	0.55	*Cry*2	*Pparγ*	0.64
*Nr*1*d*1	*Cpt*1a	0.58	*Cry*2	*Apoa*1	0.67	*Bmal*2	*Acsl*1	0.75
*Bmal*2	*Cpt*1a	0.78	*Nr*1*d*2	*Lxrα*	0.51	*Per*2	*Acsl*1	0.57
*Cry*2	*Rxra*	0.64	*Nfil*3	*Lxrα*	−0.56	*Clock*	*Acsl*1	0.53
*Bmal*2	*Rxra*	0.59	*Clock*	*Lxrα*	0.50	*Nr*1*d*2	*Pdk*4	0.55
*Per*2	*Rxra*	0.54	*Bmal*1	*Pparα*	0.52	*Cry*2	*Lipin*1	−0.54
*Clock*	*Tim*	−0.50	*Nr*1*d*1	*Pparα*	−0.54			

**Table 7:** Correlation indices between the core clock genes and the lipid metabolism genes in the liver tissue of the HFD group.

Gene	Gene	Correlation Coefficient r	Gene	Gene	Correlation Coefficient r	Gene	Gene	Correlation Coefficient r
*Bmal*1	*Cry*1	0.81	*Bmal*1	*Per*1	0.84	*Per*1	*Dbp*	0.79
*Bmal*1	*Dbp*	0.58	*Per*1	*Cry*1	0.74	*Cry*1	*Dgat*1	0.80
*Cry*1	*Hmgcr*	0.64	*Bmal*1	*Dgat*1	0.88	*Dbp*	*Dgat*1	0.51
*Bmal*2	*Hmgcr*	0.50	*Per*1	*Dgat*1	0.88	*Per*1	*Pparγ*	0.50
*Dbp*	*Pparα*	0.77	*Bmal*1	*Pparα*	0.53	*Dbp*	*Rxra*	0.70
*Per*1	*Pparα*	0.73	*Cry*1	*Pparα*	0.62	*Per*1	*Rxra*	0.50
*Cry*1	*Lxrα*	0.72	*Bmal*1	*Lxrα*	0.54	*Bmal*2	*Pparβ*	−0.52
*Per*1	*Lxrα*	0.53	*Clock*	*Lxrα*	0.56	*Bmal*2	*Ldlr*1	−0.56
*Bmal*2	*Ror* *α*	−0.75						

## Data Availability

The study did not report any other data.
